# Modified Nested Barker Codes for Ultra-Wideband Signal–Code Constructions

**DOI:** 10.3390/s23239528

**Published:** 2023-11-30

**Authors:** Vadim A. Nenashev, Aleksandr R. Bestugin, Alexey V. Rabin, Sergei V. Solenyi, Sergey A. Nenashev

**Affiliations:** Saint-Petersburg State University of Aerospace Instrumentation, 67, Bolshaya Morskaia Str., 190000 St. Petersburg, Russia; fresguap@mail.ru (A.R.B.); alexey.rabin@gmail.com (A.V.R.); ssv555ssv@yandex.ru (S.V.S.); nenashev_sergey178@mail.ru (S.A.N.)

**Keywords:** modified Barker codes, normalized autocorrelation function, nested codes, side lobe level, UWB signal–code constructions

## Abstract

Currently, various applications of ultra-wideband signal–code constructions are among the most vibrant technologies, being implemented in very different fields. The purpose of this research consists of analyzing Barker codes and searching for the optimal nested representations of them. We also aim to synthesize signal–code constructions based on the tenets of nesting of alternative modified Barker codes, which employ an asymmetric alphabet. The scientific merit of the paper is as follows: on the basis of new analytic expressions, modified nested codes and signal–code constructions were obtained, applicable for the establishment of the unambiguous association of the component values of the nested codes with any lobes of the normalized autocorrelation function. With these analytical expressions, we are, hence, able to determine the values of the binary asymmetrical components of the nested codes related to the side lobes of the normalized autocorrelation function. In this way, we clearly obtain better (low) levels for these lobes than by using the autocorrelation function, as established by the equivalent conventional Barker codes, including the nested constructions. Practical application of these modulated ultra-wideband signals ensures improved correlational features, high-fidelity probabilistic detection, and more precise positional detection of physical bodies depending on the range coordinate.

## 1. Introduction

Recently, in the domain of far-range probing system development, complex modulated signals have found wider applications. This is to solve different problems related to retrieving signals from ambient noise. To do so, a signal compression procedure in the strobe of the receiver is required. In terms of this workflow, where the signals as such are code-modulated, the workflow of modulated signal compression is implemented on the basis of correlational processing methods. Thereby, the features of the correlational reception of a compressed signal–code construction depend on the actual code within this construction [1,2,3]. However, not every code can be a template for signal–code constructions. This provides a certain workflow of retrieval (detection) of a code-modulated signal in the ambient noise. As a rule, signal–code constructions depend on the side lobe (SL) level of a normalized autocorrelation function (NACF), obtained after compression. This level has to be the minimum required for the most efficient solution of the exact detection problem.

The main applied code-modulated amplitude signals are the ultra-wideband (UWB) ones [4,5]. Hence, one of the most promising trends in the development of UWB location techniques [6], including multi-positional ones [6,7,8], consists of the batching of modulated UWB pulses, particularly in the employment of Barker codes and their modifications [9,10,11]. This is because they are suitable for UWB signal batching via hardware-based UWB amplitude polarity reversal upon generation.

Modified Barker codes, in contrast to regular codes, contain some asymmetries in certain code components and their parts. This approach, consequently, complicates the synthesis of such signals, which are phase-modulated with this code. In UWB detection devices, the compression routine for the generated signal–code constructions is implemented on the basis of the generated signal and depends on the amplitude-modulated one. Hence, the value of the positive code component corresponds to the equivalent amplitude value for the UWB signal. The same holds true for the negative part of the component and amplitude modulation of the UWB signal.

In the second part of this paper, we consider codes which are applied for amplitude modulation; specifically, the Barker codes and code sequences with the asymmetric alphabet modified upon them. According to the obtained analytical expressions characterizing the side lobe level, a search for new values of the code components is performed. Further, we present a comparative analysis of the modified variants with conventional Barker code representations to compare and contrast the SL-derived evaluations of NACF for conventional and modified Barker codes with an asymmetrical alphabet. Further, we compute the difference between these levels. Upon comparative analysis, we infer if it is viable to extend this analytical approach to the composition of nested code constructions. Thus, in the third part, we discuss the peculiarities of the generation of nested code constructions using Barker codes and derive an analytical expression to retrieve the values for code components with an asymmetrical alphabet in the vein of the research presented in the second section for particular instances of detection problems. Computational experiments are presented which are necessary for the retrieval of the numerical values of nested code components. Here, we build on Barker codes with asymmetrical alphabets. In addition, numerical characteristics are given for various levels of SL NACFs which possess the optimal correlation features. In the fourth part, we give example implementations of signal–code constructions upon the new values obtained for the components of the modified codes from the second and third parts to further implement them in actual UWB devices and their respective (including spatially distributed) systems [12]. We also present the results of our computer simulations, in which we model the compression of the generated signal–code constructions. We further give the numerical characteristics of the correlational features of these UWB signals modulated by the modified Barker codes and their respective nested constructions. Finally, in the fifth part, we conclude our research and suggest further possible applied implementations of the results and extension of their applicability in the domain.

## 2. Modified Barker Codes

Traditionally, certain approaches are used to modulate these signals [13,14] and, consequently, to achieve a high fidelity of signal detection in ambient noise. This is particularly relevant with a sequence of codes. When choosing an actual code, the following principle is in action: the best code is the one with the lowest level of SL NACF. It is known that in Barker code sequences, this level is 1/*N*, where *N*—code length. These codes are widely used in the design of various devices and detection systems, including the ultra-wideband ones. This is because they are unique binary codes with a symmetric alphabet {1, −1}, concerning the regular distribution of values at level SL {1, 0} ACF. The data on finite-length Barker codes with a symmetric alphabet and the respective SL NACF levels are presented in Table 1.

In conventional detection systems, the following workflow is usually performed: in detection systems, a fixed-length Barker sequence is selected according to the requirements for the correlational characteristic. This ensures the process of its detection in the receiver.

Apart from radar applications, Barker codes can also be employed in the systems for frame-by-frame data streaming. For example, if the code sequence is detected on the receiving end, the receiver ensures that the start of the frame is found, and further, the system performs the frame data retrieval. These data are the payload transmitted between the subscribers. The sequence identification process consists of mapping input data with a copy of the preamble, stored on the receiving end [16,17,18].

However, in actual detection systems, such as two-way subscriber lines or various ranging systems, the correlational approach is used.

To further illustrate this approach, we consider the case of conventional ACF computation for the maximum-length Barker code *N* = 13. For other codes, the workflow is effectively the same.

**Figure 1:**
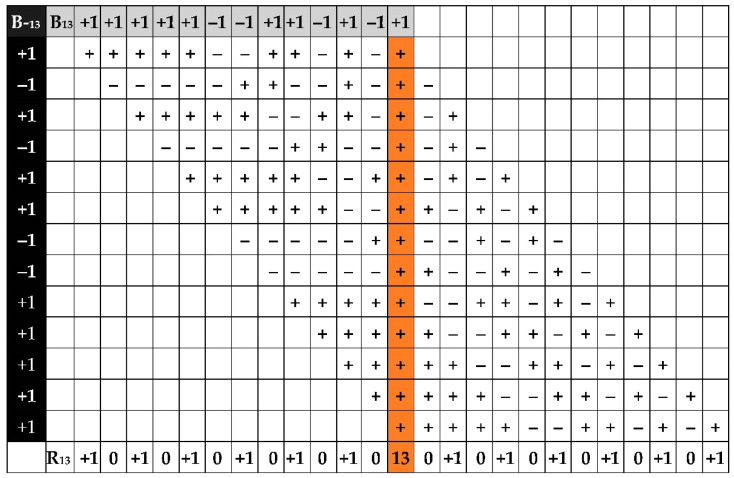
The workflow of conventional computation of the values for levels SL ACF for the Barker code *N* = 13.

The workflow of conventional computation of the values for levels SL ACF for the Barker code *N* = 13 is presented in Figure 1.

For the length of conventional Barker code *N* = 13, the following ACF values were computed: *R*_13_ = {1, 0, 1, 0, 1, 0, 1, 0, 1, 0, 1, 0, 13, 0, 1, 0, 1, 0, 1, 0, 1, 0, 1, 0, 1}.

However, this workflow is not applicable for obtaining analytical expressions for Barker codes, depending on *b*, where *b* is a negative value.

In [19], analytical expressions are presented to search for different code pairs {1, −*b*} for lengths *N* = 3, 5, 7, 11, 13. This allows extending the class of code sequences and augment the coding theory and the methods of digital complex processing of echo signals. It also enables the discovery of new code structures with superior correlational characteristics compared to the known ones. To that end, the ACF characteristics were evaluated. We then conducted a comparative analysis, contrasting the newly obtained modified code sequences with the Barker codes of varying lengths considered in this study.

The analytical expressions that enable the search for b the new modified components of Barker codes are presented in Table 2. In contrast to [19], these expressions are augmented with new terms for lengths *N* = 2 and 4.

In [19], research efforts were summarized with the aim of searching for modified code values for the pair {1, −*b*}. This pair considers two values simultaneously: one for the positive and one for the negative level of SL ACF. However, these analytical expressions (see Table 2) enable the calculation of negative code value, denoted as —*b*, which is relevant for solving a specific detection problem [20].

This scenario arises when it is necessary to evaluate the extremum values for both positive and negative SL NACF values simultaneously. Simultaneous evaluation becomes feasible in the implementation of a detection system, especially in the decimeter scale of a ranging system when measuring the levels of SL NACF.

Table 3 presents the results of the evaluation of SL levels for a conventional Barker code and various code modifications. Additionally, we computed the level difference between the conventional and modified codes.

Having obtained the values SL ACF depending on the component pair of the code {1, −*b*}, we present the resulting evaluations of SL NACF with the optimal value *b* in Table 3.

Hence, it follows from the evaluation results, presented in Table 3, that the best characteristic is associated with the modified Barker code of length *N* = 13, exhibiting a maximum SL NACF of −23.77 dB, where the code component value is *b =* 3 −$\sqrt{3}$ in the pair {1, −*b*}.

Transitioning to the modulation of signals tagged with various asymmetric pair {1, −*b*}, as opposed to the known symmetric pair {1, −1}, it is possible to ensure the elimination of any ambiguities in their retrieval. This further enables accommodation, for example, for target audience growth when using a distributed remote probing system that employs different inspection angles.

Deviation from conventional symmetric representations of codes, coupled with the use of modified asymmetric pairs, allows for the extension of the class of code sequences, the augmentation of coding theory, and the exploration of novel methods for generating modulated signals based on such codes, whose characteristics surpass those of the known ones.

However, the results presented in Table 3 may not always ensure the required correlational characteristics when implementing an actual detection system relying on the SL NACF level. This limitation arises because the length of such codes cannot exceed *N* = 13 [21].

Therefore, we propose extending this approach to codes longer than Barker codes. To achieve this, we employed the code nesting approach and explored various possible combinations of Barker codes.

## 3. The Analysis of Side Lobe Level in Modified Nested Barker Code Constructions, Having an Asymmetrical Alphabet

As demonstrated earlier, the maximum effective (SL) level values achievable with Barker codes and their modified counterparts, utilizing an asymmetrical alphabet, are −22.28 dB and −23.77 dB, respectively, representing a difference of 1.49 dB. However, this result does not always meet the requirements of modern small-sized radar stations. Nevertheless, Barker codes and their modifications can be amalgamated into nested code constructions to generate codes with a length exceeding 13. In this case, a *B_M_* code can be used as *B_N_* in the code (*M* within *N*) to generate the code *B_M_*_×*N*_ of length *M* × *N*.

**Figure 2:**
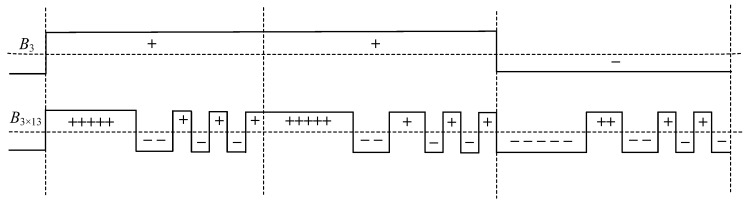
Barker code construction 3 × 13 (above) and signal envelope for this code (below).

As an example, consider a code construction of length *B*_3×13_, structured as follows: *B*_3×13_ = {+ + + + + − − + + − + − +, + + + + + − − + + − + − +, − − − − − + + − − + − + −} and shown in Figure 2.

**Figure 3:**
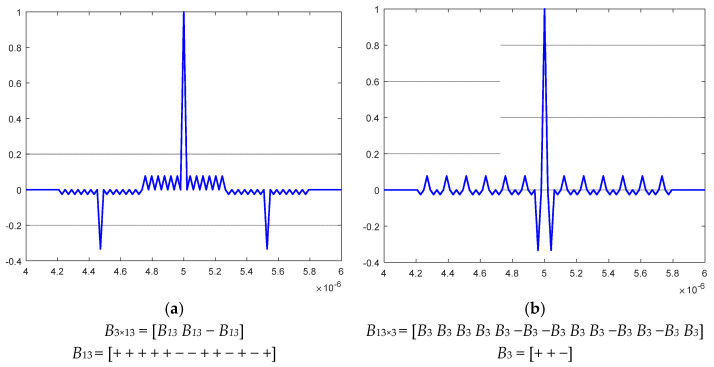
NACF of a nested Barker code construction with a Length of 39—*B*_3×13_ combined of respective 13-component and 3-component conventional code representations.

In Figure 3, NACFs for nested Barker codes [22,23] are presented, where the combined length is *N* = 39:

Figure 3a presents an NACF of a nested Barker code construction with a length of 39—*B*_3×13_. The graph displays two prominent negative SL peaks with a value of −13/39, and the maximum positive SL peaks of the NACF, reaching a level of 3/39.

Figure 3b presents an NACF of a nested Barker code construction with a length of 39—*B*_13×3_ of the second type. Similar to Figure 3a, it showcases the largest positive and negative values in the SL levels of the NACF but in different spatial locations.

Furthermore, we delve into the analysis of the SL NACF levels for the construction of nested codes generated based on modified Barker codes.

### 3.1. Nested Code Constructions Based on the Modified Barker Codes

Due to the significantly higher level of SL NACF observed in the discovered modified Barker codes with an asymmetrical alphabet compared to conventional Barker codes with a symmetrical alphabet, they can be effectively employed to construct new nested code sequences.

In contrast to Barker codes and their modifications, code constructions based on them can have several different implementations. Sequences composed exclusively of conventional Barker codes with a symmetrical alphabet are denoted as “Barker-Barker” (BB), while those composed exclusively of modified variants with an asymmetric alphabet are denoted as “Modification-Modification” (MM). Two hybrid compositions of code constructions are also possible. The first involves a nested modified Barker code being “multiplied” using the Kronecker product by the components of a conventional Barker code, resulting in the configuration “Barker-Modification” (BM). The second, symmetrical one is established where a nested Barker code is “multiplied” by the components of a modified Barker code—"Modification-Barker” (MB). Therefore, four various compositions of code constructions should be considered: BB, BM, MB, MM, and the best among them needs to be determined. Additionally, it is essential to find a component *b* of code constructions that enables the variants BM, MB, and MM to outperform the conventional BB variant.

Table 4 presents different variants of code constructions: BB, BM, MB, and MM, each with a length of 13 × 3.

Table 5, Table 6, Table 7 and Table 8 present the results of the maximum positive SL level evaluation for various code constructions, each falling into one of four variants: BB, BM, MB, and MM. Additionally, we present the calculated differences between the following estimates: “BB-BM”, “BB-MB”, and “BB-MM”. The last column, labeled “best type”, indicates the nested code structure with the lowest level of SL NACF compared to other code structures of the same length. Estimates of SL NACF levels are presented in two dimensions, relative and in dB.

The value of the *b* component for modified codes (“BM”, “MB”, and “MM”) was selected in accordance with Table 3. Given that the retrieval of a compressed echo-signal is a time-dependent process, the detector features are primarily influenced by the SL level of an NACF.

As evident from the data provided in Table 5, Table 6, Table 7 and Table 8, the modified nested code structures of the “BM”, “MB”, and “MM” types exhibit superior characteristics compared to similar designs of the classical symmetrical “BB” type for various lengths and combinations with type-based distribution:

− “BB, BM”: 4 × 3, 4 × 5, 4 × 7 and 4 × 13;
− “BM”: 2 × 3, 3 × 3, 3 × 5, 3 × 13, 7 × 3, 7 × 13, 11 × 3, 11 × 5, 11 × 13 and 13 × 5;
− “MB”: 3 × 4, 5 × 2, 5 × 11, 7 × 4, 13 × 2, 13 × 7 and 13 × 11;
− “MB, MM”: 5 × 3, 5 × 7, 5 × 13 and 13 × 3;
− “MM”: 5 × 5, 7 × 5 and 13 × 13.

This result allows us to draw conclusions about the feasibility and prospects of using modified codes for certain lengths in modern detection systems, particularly in ultra-wideband ones. However, this approach, despite exhibiting lower characteristics for the mentioned nested code structures compared to their classical symmetric representations, lacks analytical expressions suitable for determining the values of elements in nested codes. This limitation prevents us from identifying the best values for the SL NACF estimate for the modified nested code at different lengths. Consequently, the optimal characteristics continue to favor the “BB” type for the following lengths: 2 × 2, 2 × 4, 2 × 5, 2 × 7, 2 × 11, 2 × 13, 3 × 2, 3 × 7, 3 × 11, 4 × 2, 4 × 4, 4 × 11, 5 × 4, 7 × 2, 7 × 7, 7 × 11, 11 × 2, 11 × 4, 11 × 7, 11 × 11, 13 × 4.

### 3.2. The Search for Optimal Values of Nested Code Components via Their Analytical Expressions

Furthermore, we will illustrate the workflow of a value search for nested code constructions using analytical expressions, exemplified by a code of length 3 × 3.

To illustrate how to search for the components of nested code constructions, we need to represent the modified Barker codes in two variants: substituting the negative part by *b*_1_ in the first instance and by *b*_2_ in the second instance, as follows: M_3*a*_ = [1, 1, *b*_1_] and M_3*b*_ = [1, 1, *b*_2_]. If we were to substitute the negative part by *b* only in one variant, M_3_ = [1, 1, *b*], then the search for optimal values would be precluded because some analogous components of the modified codes could differ from each other.

This limitation is evident in the resulting analytical expressions provided in Table 9, which are used to determine the values of lobe levels in ACF.

Let us delve into the analytical expressions that incorporate the values *b*_1_ and *b*_2_. Upon solving these expressions, we derived a modified code pair with values: M_3*a*_ = [1, 1, −1.721] and M_3*b*_ = [1, 1, 0.1211], where the maximum SL level of an NACF was found to be R_max+_ = −0.0209. We denote this type of code construction as MM2.

By comparison, a nested construction of type BM with values {1, −1} × {1, −0.5} yielded an equivalent value of 0.0741. Consequently, analytical expressions that incorporate values *b*_1_ and *b*_2_ and define the level values for ACF lobes allow us to find superior values for components of nested codes compared to those considered in previous sections. For instance, the difference between a nested code of length 3 × 3 of type MM2 compared to an equivalent code of type BB (i.e., BM−MM2) is 0.0950. Therefore, based on this result, nested codes of type MM2 with various values *b*_1_ and *b*_2_ are superior to the initial ones in terms of the maximum level of SL NACF, outperforming BM and particularly surpassing instances of the conventional BB type.

Similar research was also conducted for other lengths of nested codes for constructions of type MM2.

Thus, the methodology for finding negative values [*b*_1_, *b*_2_] for modified nested Barker codes is as follows:

(1) Select a nested Barker code sequence of desired length *N*×*M* with values in the pair {1, −1}.

(2) Plot the NACF graph for the selected nested Barker code sequence of length *N*×*M* in step 1.

(3) Determine the maximum SL value of the NACF obtained in step 2.

(4) In the nested code selected for research in step 1, replace the negative elements of the code with the value −1 with [*b*_1_, *b*_2_], thus defining a nested code of type MM2: {1, *b*_1_}×{1, *b*_2_}.

(5) Obtain expressions for each lobe (main and side) of the ACF as a function of *b*_1_ and *b*_2_.

(6) Obtain expressions for each SL of the NACF, thus forming a system of expressions.

(7) Find the parameters *b*_1_ and *b*_2_ at which the value of SL NACF will be the smallest possible by solving the corresponding system of expressions obtained in the previous step.

(8) Verify that the values of NACF SL levels at the found values of *b*_1_ and *b*_2_ in the previous step are lower than the NACF SL level determined in step 3.

Table 10 presents the results of such research, including values for components of the “best type” of nested codes (see Table 5, Table 6, Table 7 and Table 8) and MM2. Here, the evaluations of maximum SL level are provided in terms of NACF, as well as the difference in this value from the SL level for nested codes of the “best type” data from Table 5, Table 6, Table 7 and Table 8.

The analysis of numerical data from Table 10 shows that by employing the workflow to search for two component values belonging to the code of type MM2 based on the respective analytical values, it is possible to downgrade the level of SL NACF in comparison with the SL of the best types given in Table 5, Table 6, Table 7 and Table 8.

For example, for a nested code structure of a length 13 × 13 with values *b*_1_ = −1.6 and *b*_2_ = −2, the difference between MM and MM2 was 0.0163.

The remaining numerical results also demonstrated a decrease in the level of SL NACF in the case of using the values of *b*_1_ and *b*_2_ obtained from the corresponding analytical expressions for modifying the nested code structure of the MM2 type, with the exception of lengths 2 × 2 and 11 × 11.

This leads us to a conclusion that it is appropriate to use a new modification of nested codes (type MM2) with newfound values of negative components *b*_1_ and *b*_2_. Obtained with the derived analytical expressions, this enables the optimal correlation data features of the nested code constructions.

## 4. The Generation of Conventional and Modified Ultra-Wideband Signal–Code Constructions and Their Nested Representations

In Section 2, modified Barker codes with optimal SL NACF level were obtained. This research, based on numerical simulations, demonstrated that introducing some asymmetry into the alphabet of a binary Barker code could significantly improve the ratios of side lobes and the main ACF peak. Further, examples of modified Barker codes in action, as well as their representations, are required. These examples should be applied in the modulation (synthesis) of probing signals. Since it is not always possible to enhance the mathematical description, the modification of Barker codes and their nested representations can find applications in actual devices and detection systems.

Hence, the most obvious application of the discovered modified Barker codes (see Section 2) and their respective nested code constructions (see Section 3) is their implementation in physical devices and detection systems to generate probing signals. The key approach here is an amplitude modulation of UWB signals, including the ones with various asymmetric values of pulse levels [24,25,26].

Currently, the implementation of ultra-wideband signals is among the most novel and promising technologies, finding applications in very different industries.

It is known that one of the most promising extensions of UWB ranging methods [27,28], including multi-positional ones [29,30,31,32], is the batched application of encoded UWB pulses, particularly the implementation of Barker codes. These codes enable the composition of UWB batches by changing polarity, and hence, the amplitude of an elementary pulse. Here, a positive code component has an amplitude polarity “+”, and a negative one, correspondingly, “−” [33].

Additionally, the analysis of the current advances in wireless technologies, employing the amplitude modulation of ultra-wideband signals, is acknowledged to be promising in the further development of digital processing of similar signal–code constructions in difficult-to-handle noisy environments [34,35,36,37].

The average power of a UWB signal is particularly low due to its ultra-short pulses, specifically from 0.5 to 2 ns. Therefore, employing the amplitude modulation of UWB signals is practical to increase their average power in ultra-wideband detection systems. Generating an UWB signal–code design is necessary to detect inhomogeneities at short distances, especially in solid media (soil, concrete, etc.) [26].

Furthermore, we need to examine examples of generation elementary UWB pulses, modulated by modified codes described in Section 2 and code constructions described in Section 3. We will then compare and contrast them with equivalent signals modulated by conventional codes. This evaluation is necessary to assess the features of compressed code-modulated signals, particularly the SL level of an NACF.

**Figure 4:**
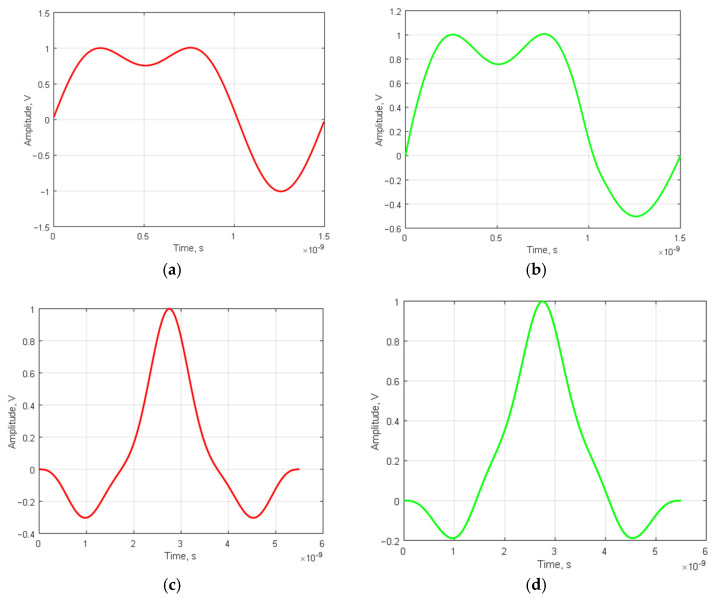
UWB-signals, amplitude-modulated by a conventional (**a**) B_3_ = [1, 1, −1] and a modified (**b**) Barker code M_3_ = [1, 1, −0.5] along with their respective NACFs (**c**,**d**), are presented: (**a**) UWB signal modulated by the classical Barker code, length *N* = 3; (**b**) UWB signal modulated with a modified Barker code, length *N* = 3; (**c**) NACF of a UWB signal modulated with a classical Barker code of length *N* = 3; (**d**) NACF of a UWB signal modulated with a modified Barker code of length *N* = 3.

In Figure 4, we present UWB signals, which are amplitude-modulated by a conventional symmetric Barker code and by a modified asymmetric one.

For the same signal modulated by a modified Barker code of length 3, the resulting SL ACF value was −0.2222, while for the one modulated by a conventional Barker code of equal length, it was 0.3333. Therefore, the conventional UWB Barker signals are inferior to the modified signals by 0.1111. These results align with the theoretical ones shown in Table 3.

As seen in Figure 4c,d, the width of the main peak is practically the same for the UWB signal modulated by the canonical Barker code and its modification. To narrow the width of the ACF main lobe, optimization methods, as provided in [38,39,40], can be applied.

Therefore, both Barker code sequences and their modifications can be employed for the generation of UWB signals.

**Figure 5:**
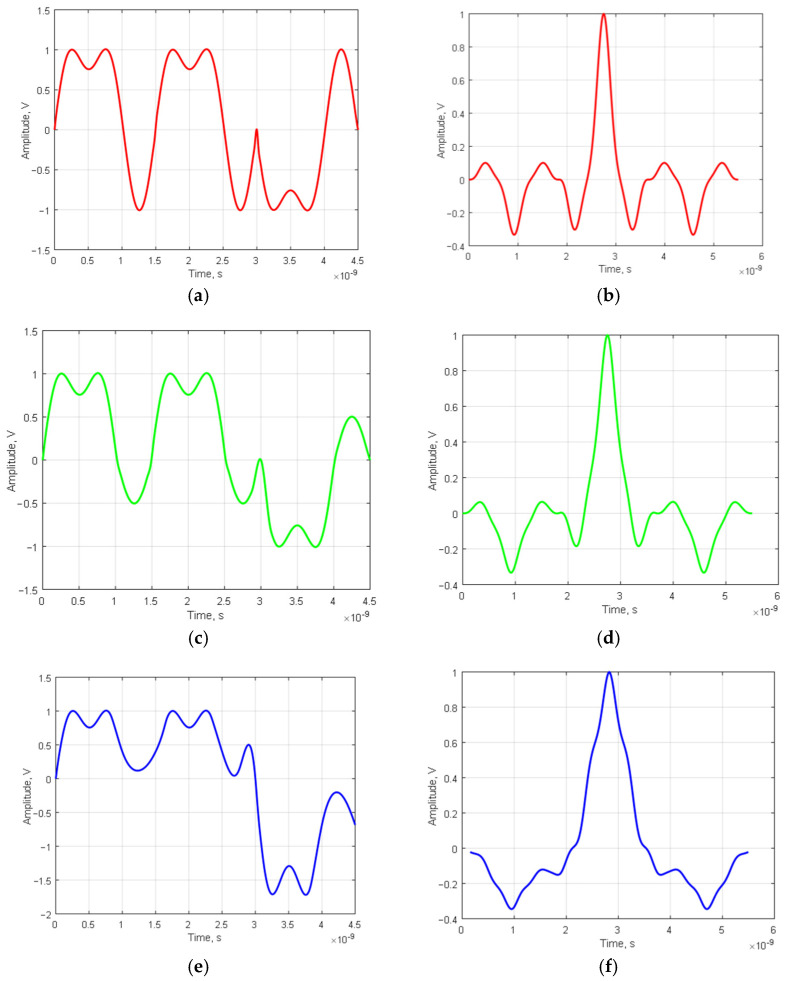
Signal–code constructions, modulated by amplitude with a conventional Barker code (**a**) and modified Barker codes (**c**,**e**), along with their respective NACFs (**b**,**d**,**f**): (**a**) UWB signal modulated with an embedded code of the BB type, length 3 × 3 (**b**) NACF of a UWB signal modulated by a classical Barker code of length 3 × 3; (**c**) UWB signal modulated with embedded BM type code, length 3 × 3; (**d**) NACF of a UWB signal modulated with a modified Barker code of length 3 × 3; (**e**) UWB signal modulated with an embedded code of type MM2, length 3 × 3; (**f**) NACF of a UWB signal modulated with a modified Barker code of length 3 × 3.

Furthermore, we need to investigate the compression characteristics of ultra-wideband signals by modified Barker codes and their signal–code constructions. The results for nested codes of types BB, BM, and MM2 (length 3 × 3) are presented in Figure 5.

The results (see Figure 5) for UWB signal–code nested structures align with the theoretical ones provided in Table 10, consistent with previous results (see Figure 4), which were in accordance with the estimates in Table 3.

For the other lengths and types of signal–code constructions, the maximum SL ACF levels were evaluated, and the resulting scores are consistent with the codes presented in Table 3, Table 8 and Table 10.

The results obtained in this research, after the performed analysis, demonstrate rich possibilities, arising from code asymmetry and relaxed requirements for the ACF. This research aims to stimulate the further exploration of algorithms of signal generation and processing to ensure more reliable signal retrieval in challenging (natural) noisy environments. This is particularly relevant for channels probing UWB signals.

Therefore, the present research holds significant practical importance in the domain of UWB systems for detecting amplitude-modulated signals, where analog, not digital, processing approaches are used.

## 5. Conclusions

This research aimed to explore new noise-proof (noise-resistant) codes, code combinations, ultra-wideband signal–code constructions, as well specialized approaches for their processing in modern UWB radar-based detection systems. These alternatives can be considered to conventional codes already applied in industry, offering improved correlation features compared to known codes and UWB-signals generated using them.

The primary outcome of this research, motivating further development in coding theory, involves deviating from the existing pairwise nested code representations, such as {1, −1} × {1, −1}, and instead using {1, *b*_1_} × {1, *b*_2_}. The obtained results differ from previously published ones by introducing, for the first time, a system of analytical expressions depending on [*b*_1_, *b*_2_] to calculate the levels of side lobes of the normalized ACF. Solving this system according to the defined new method (Section 3.2) enables finding negative elements for the nested code, minimizing the maximum level of side lobes of the normalized ACF compared to canonical representations, as presented in Table 10.

From a practical standpoint, Section 4 demonstrates that the proposed modifications to nested codes significantly enhance the functionality of measuring ultra-wideband radar devices by generating UWB signal–code structures. Examples of generating and compressing UWB signal–code structures modulated by new modified nested codes underscore their feasibility when compared to canonical analogous Barker signals.

The potential of this research lies in extending the application of the developed new methodology to search for modifications for various other types of codes (M-sequences, Frank codes, etc.) to generate signal–code structures in radar, acoustic, optical, hydroacoustic, and various multi-position remote sensing systems. Additionally, these modifications can be applied to ensure the reliability of information transmission in telecommunication communication channels.

## Figures and Tables

**Table 1:** Sequences of Barker codes and SL NACF levels *N* = 3 [15].

Code Length, *N*	Barker Sequence with Symmetric Alphabet		SL NACF Levels in dB	SL NACF Levels	
2	+1 −1	or +1 +1	−6.02	−1/2	1/2
3	+1 +1 −1		−9.54	−1/3	
4	+1 +1 −1 +1	or +1 +1 +1 −1	−12.04	±1/4	
5	+1 +1 +1 −1 +1		−13.98	1/5	
7	+1 +1 +1 −1 −1 +1 −1		−16.90	−1/7	
11	+1 +1 +1 −1 −1 −1 +1 −1−1 +1 −1		−20.83	−1/11	
13	+1 +1 +1 +1 +1 −1 −1 +1 +1 −1 +1 −1 +1		−22.28	1/13	

**Table 2:** Analytical expressions that enable the search for *b* the new modified components of Barker codes.

Lobe Number ACF	*N* = 2	*N* = 3	*N* = 4	*N* = 5	*N* = 7	*N* = 11	*N* = 13
*R*_1_	*b*^2^ + 1	*b*^2^ + 2	*b*^2^ + 3	*b*^2^ + 4	3*b*^2^ + 4	6*b*^2^ + 5	4*b*^2^ + 9
*R*_2_	−*b*	−*b* + 1	−2*b* + 1 or −*b* + 2	−2*b* + 2	*b*^2^ − 3*b* + 2	3*b*^2^ − 5*b* + 2	*b*^2^ − 6*b* + 5
*R*_3_		−*b*	−*b* + 1	−*b* + 2	*b*^2^ − 3*b* + 1	3*b*^2^ − 5*b* + 1	*b*^2^ − 5*b* + 5
*R*_4_			1 or −*b*	−*b* + 1	(*b* − 1)^2^	3*b*^2^ − 4*b* + 1	*b*^2^ − 5*b* + 4
*R*_5_				1	−2*b* + 1	2*b*^2^ − 4*b* + 1	(*b* − 2)^2^
*R*_6_					−*b* + 1	2*b*^2^ − 3*b* + 1	*b*^2^ − 4*b* + 3
*R*_7_					−*b*	*b*^2^ − 3*b* + 1	*b*^2^ − 3*b* + 3
*R*_8_						(*b* − 1)^2^	−3*b* + 3
*R*_9_						−2*b* + 1	−2*b* + 3
*R*_10_						−*b* + 1	−2*b* + 2
*R*_11_						−*b*	−*b* + 2
*R*_12_							−*b* + 1
*R*_13_							1

**Table 3:** ACF values of the modified codes.

Code Length, *N*	Estimating Code Parameters			
	The Value of −*b* for the Modified Barker Pair {1, −*b*}	SL Level of the Classical Barker Code	SL Level of Modified Barker Code	Difference between Classic and Modified
2	−1	−6.02 dB	-	-
3	−0.5	−9.54 dB	−13.06 dB	3.52 dB
4	−1	−12.04 dB	-	-
5	−1.5	−13.98 dB	−15.92 dB	1.94 dB
7	$\sqrt{2}-2$	−16.90 dB	−18.68 dB	1.78 dB
11	−1	−20.83 dB	-	-
13	$\sqrt{3}-3$	−22.28 dB	−23.77 dB	1.49 dB

**Table 4:** Different variants of code constructions 13 × 3.

Barker Code View	Barker Code
BB	[{1, 1, −1}, {1, 1, −1}, {1, 1, −1}, {1, 1, −1}, {1, 1, −1}, {−1, −1, 1}, {−1, −1, 1}, {1, 1, −1}, {1, 1, −1}, {−1, −1, 1}, {1, 1, −1}, {−1, −1, 1}, {1, 1, −1}]
BM	[{1, 1, −*b*}, {1, 1, −*b*}, {1, 1, −*b*}, {1, 1, −*b*}, {1, 1, −*b*}, {−1, −1, *b*}, {−1, −1, *b*}, {1, 1, −*b*}, {1, 1, −*b*}, {−1, −1, *b*}, {1, 1, −*b*}, {−1, −1, *b*}, {1, 1, −*b*}]
MB	[{1, 1, −1}, {1, 1, −1}, {1, 1, −1}, {1, 1, −1}, {1, 1, −1}, {−*b*, −*b*, *b*}, {−*b*, −*b*, *b*}, {1, 1, −1}, {1, 1, −1}, {−*b*, −*b*, *b*}, {1, 1, −1}, {−*b*, −*b*, *b*}, {1, 1, −1}]
MM	[{1, 1, −*b*}, {1, 1, −*b*}, {1, 1, −*b*}, {1, 1, −*b*}, {1, 1, −*b*}, {−*b*, −*b*, *b*^2^}, {*b*, −*b*, *b*^2^}, {1, 1, −*b*}, {1, 1, −*b*}, {−*b*, −*b*, *b*^2^}, {1, 1, −*b*}, {−*b*, −*b*, *b*^2^}, {1, 1, −*b*}]

**Table 5:** SL NACF level evaluation for nested BB type codes (without modification).

Code Length, *N* × *M*	BB	Best Type	Code Length, *N* × *M*	BB	Best Type	Code Length, *N* × *M*	BB	Best Type
2 × 2	0.25	BB	4 × 2	0.2500	BB	11 × 2	0.0455	BB
	−12.0412			−12.0412			−26.8485	
2 × 4	0.3750	BB	4 × 4	0.3125	BB	11 × 4	0.2500	BB
	−8.5194			−10.1030			−12.0412	
2 × 11	0.0455	BB	4 × 11	0.25	BB	11 × 11	0.0083	BB
	−26.8485			−12.0412			−41.6557	

**Table 6:** SL NACF level evaluation for nested BB and BM type codes.

Code Length, *N* × *M*	BB	BM	Differences between BB-BM	Best Type	Code Length, *N* × *M*	BB	BM	Differences between BB-BM	Best Type
2 × 3	0.1667	0.1111	0.0556	BM	4 × 7	0.25	0.25	0	BB, BM
	−15.5630	−19.0849	−3.5219			−12.0412	−12.0412	0	
2 × 5	0.2000	0.2400	−0.04	BB	4 × 13	0.25	0.25	0	BB, BM
	−13.9794	−12.3958	1.5836			−12.0412	−12.0412	0	
2 × 7	0.0714	0.1235	−0.0521	BB	11 × 3	0.0303	0.0202	0.0101	BM
	−22.9226	−18.1667	4.7559			−30.3703	−33.8930	−3.5227	
2 × 13	0.0769	0.0972	−0.0203	BB	11 × 5	0.2000	0.1600	0.04	BM
	−22.2789	−20.2459	2.033			−13.9794	−15.9176	−1.9382	
4 × 3	0.25	0.25	0	BB, BM	11 × 7	0.0130	0.0824	−0.0694	BB
	−12.0412	−12.0412	0			−37.7298	−21.6815	16.0483	
4 × 5	0.25	0.25	0	BB, BM	11 × 13	0.0769	0.0648	0.0121	BM
	−12.0412	−12.0412	0			−22.2789	−23.7678	−1.4889	

**Table 7:** SL NACF level evaluation for nested BB and MB type codes.

Code Length, *N* × *M*	BB	MB	Differences between BB-MB	Best Type	Code Length, *N* × *M*	BB	MB	Differences between BB-MB	Best Type
3 × 2	0.1667	0.2222	−0.0555	BB	7 × 2	0.0714	0.1165	−0.0451	BB
	−15.5630	−13.0643	2.4987			−22.9226	−18.6756	4.247	
3 × 4	0.2500	0.2222	0.0278	MB	7 × 4	0.25	0.2209	0.0291	MB
	−12.0412	−13.0643	−1.0231			−12.0412	−13.1168	−1.0756	
3 × 11	0.0303	0.2222	−0.1919	BB	7 × 11	0.0130	0.1165	−0.1035	BB
	−30.3703	−13.0643	17.306			−37.7298	−18.6756	19.0542	
5 × 2	0.2000	0.1600	0.04	MB	13 × 2	0.0769	0.0648	0.0121	MB
	−13.9794	−15.9176	−1.9382			−22.2789	−23.7678	−1.4889	
5 × 4	0.25	0.2900	−0.04	BB	13 × 4	0.25	0.2662	−0.0162	BB
	−12.0412	−10.7520	1.2892			−12.0412	−11.4958	0.5454	
5 × 11	0.2000	0.1600	0.04	MB	13 × 11	0.0769	0.0648	0.0121	MB
	−13.9794	−15.9176	−1.9382			−22.2789	−23.7678	−1.4889	

**Table 8:** SL NACF level evaluation for nested BB, BM, MB, and MM type codes.

Code Length, *N* × *M*	BB	BM	MB	MM	Differences between BB-MB	Differences between BB-MB	Differences between BB-MM	Best Type
3 × 3	0.1111	0.0741	0.2222	0.2222	0.037	−0.1111	−0.1111	BM
	−19.0849	−22.6036	−13.0643	−13.0643	−3.5187	6.0206	6.0206	
3 × 5	0.2000	0.1600	0.2222	0.2222	0.04	−0.0222	−0.0222	BM
	−13.9794	−15.9176	−13.0643	−13.0643	−1.9382	0.9151	0.9151	
3 × 7	0.0476	0.0824	0.2222	0.5742	−0.0348	−0.1746	−0.5266	BB
	−26.4444	−21.6815	−13.0643	−4.8188	4.7629	13.3801	21.6256	
3 × 13	0.0769	0.0648	0.2222	0.2222	0.0121	−0.1453	−0.1453	BM
	−22.2789	−23.7678	−13.0643	−13.0643	−1.4889	9.2146	9.2146	
5 × 3	0.2000	0.2000	0.1600	0.1600	0	0.04	0.04	MB, MM
	−13.9794	−13.9794	−15.9176	−15.9176	0	−1.9382	−1.9382	
5 × 5	0.2000	0.2000	0.2000	0.1856	0	0	0.0144	MM
	−13.9794	−13.9794	−13.9794	−14.6284	0	0	−0.649	
5 × 7	0.2000	0.2000	0.1600	0.1600	0	0.04	0.04	MB, MM
	−13.9794	−13.9794	−15.9176	−15.9176	0	−1.9382	−1.9382	
5 × 13	0.2000	0.2000	0.1600	0.1600	0	0.04	0.04	MB, MM
	−13.9794	−13.9794	−15.9176	−15.9176	0	−1.9382	−1.9382	
7 × 3	0.0476	0.0318	0.5387	0.1165	0.0158	−0.4911	−0.0689	BM
	−26.4444	−29.9515	−5.3726	−18.6735	−3.5071	21.0718	7.7709	
7 × 5	0.2000	−0.1600	0.2000	0.1414	0.04	0	0.0586	MM
	−13.9794	−15.9176	−13.9794	−16.9932	−1.9382	0	−3.0138	
7 × 7	0.0204	0.0824	0.1165	0.1165	−0.062	−0.0961	−0.0961	BB
	−33.8039	−21.6815	−18.6756	−18.6756	12.1224	15.1283	15.1283	
7 × 13	0.0769	0.0648	0.1165	0.1165	0.0121	−0.0396	−0.0396	BM
	−22.2789	−23.7678	−18.6756	−18.6756	−1.4889	3.6033	3.6033	
13 × 3	0.0769	0.0769	0.0648	0.0648	0	0.0121	0.0121	MB, MM
	−22.2789	−22.2815	−23.7678	−23.7678	−0.0026	−1.4889	−1.4889	
13 × 5	0.2000	0.1600	0.2000	0.1704	0.04	0	0.0296	BM
	−13.9794	−15.9176	−13.9794	−15.3722	−1.9382	0	−1.3928	
13 × 7	0.0769	0.0824	0.0648	0.0877	−0.0055	0.0121	−0.0108	MB
	−22.2789	−21.6815	−23.7678	−21.1400	0.5974	−1.4889	1.1389	
13 × 13	0.0769	0.0769	0.0769	0.0690	0	0	0.0079	MM
	−22.2789	−22.2789	−22.2789	−23.2224	0	0	−0.9435	

**Table 9:** ACF lobes expressed analytically.

ACF Petal Number	Expressions for Searching *b* in the Case of the MM Option	Expressions for Searching *b_1_* and *b_2_* in the Case of the MM Option
*R*_1_	*b*^4^ + 4*b*^2^ + 4	*b*_1_^2^*b*_2_^2^ + 2*b*_1_^2^ + 2*b*_2_^2^ + 4
*R*_2_	*b*^3^ + 2*b*^2^ + 3*b* + 2	3*b*_2_ + *b*_1_*b*_2_ + *b*_1_^2^*b*_2_ + *b*_1_^2^ + 2
*R*_3_	*b*^3^ + *b*^2^ + 4*b* + 1	*b*_1_ + 3*b*_2_ + *b*_1_*b*_2_ + *b*_1_^2^*b*_2_ + 1
*R*_4_	*b*^3^ + *b*^2^ + 2*b* + 2	2*b*_1_ + *b*_1_*b*_2_^2^ + *b*_2_^2^ + 2
*R*_5_	2*b*^2^ + 2*b* + 1	*b*_1_ + *b*_2_ + 2*b*_1_*b*_2_ + 1
*R*_6_	*2b^2^* + 2*b*	*b*_1_ + *b*_2_ + 2*b*_1_*b*_2_
*R*_7_	*b*^3^ + 2*b*	*b*_1_*b*_2_^2^ + 2*b*_1_
*R*_8_	*b*^2^ + *b*	*b*_1_ + *b*_1_*b*_2_
*R*_9_	*b* ^2^	*b*_1_ *b*_2_

**Table 10:** Resulting scores of SL NACF levels for nested codes.

Code Length, *N* × *M*	Meaning [*b*_1_, *b*_2_] in Nested Type Code MM2: {1, *b*_1_} × {1, *b*_2_}	Maximum SL Level of NACF for Types BB/BM/MB/MM and MM2	Difference “Best Type” of Table 5, Table 6, Table 7 and Table 8	Best Type	Code Length, *N* × *M*	Meaning [*b*_1_, *b*_2_] in Nested Type Code MM2: {1, *b*_1_} × {1, *b*_2_}	Maximum SL Level of NACF for Types BB/BM/MB/MM and MM2	Difference “Best Type” of Table 5, Table 6, Table 7 and Table 8	Best Type
2 × 2	BB {1, −1} × {1, 1} MM2 {1, −1} × {1, 1}	−0.25 −0.25	0	BB	5 × 7	MM {1, −1.5} × {1, −0.5858} MM2 {1, −10} × {1, −1}	0.1600 0.0247	0.1353	MM2
2 × 3	BM {1, −1} × {1, −0.5} MM2 {1, −1} × {1, 0.5}	0.1111 −0.1111	0.2222	MM2	5 × 11	MB {1, −1.5} × {1, −1} MM2 {1, −10} × {1, −1}	0.1600 0.0157	0.1443	MM2
2 × 4	BB {1, −1} × {1, −1} MM2 {1, 0} × {1, −2}	0.3750 0.1429	0.2321	MM2	5 × 13	MM {1, −1.5} × {1, −1.2679} MM2 {1, −10} × {1, −2}	0.1600 0.0608	0.0992	MM2
2 × 5	BB {1, −1} × {1, −1} MM2 {1, 0} × {1, −2}	0.2000 0.1250	0.0750	MM2	7 × 2	BB {1, −1} × {1, −1} MM2 {1, −1} × {1, −10}	0.0714 0.0141	0.0573	MM2
2 × 7	BB {1, −1} × {1, −1} MM2 {1, 0} × {1, −1}	0.0714 0	0.0714	MM2	7 × 3	BM {1, −1} × {1, −0.5} MM2 {1, −1} × {1, −10}	0.0318 0.0140	0.0178	MM2
2 × 11	BB {1, −1} × {1, −1} MM2 {1, 0} × {1, −1}	0.0455 0	0.0455	MM2	7 × 4	MB {1, −0.5858} × {1, −1} MM2 {1, −1} × {1, −10}	0.2209 0.0264	0.1945	MM2
2 × 13	BB {1, −1} × {1, −1} MM2 {1, 0} × {1, −2}	0.0769 0.0376	0.0393	MM2	7 × 5	MM {1, −0.5858} × {1, −1.5} MM2 {1, −1} × {1, −10}	0.1414 0.0247	0.1167	MM2
3 × 2	BB {1, −1} × {1, −1} MM2 {1, −2} × {1, 1}	0.1667 −0.1667	0.3334	MM2	7 × 7	BB {1, −1} × {1, −1} MM2 {1, −0.89} × {1, −0.89}	0.0204 0.0198	0.0006	MM2
3 × 3	BM {1, −1} × {1, −0.5} MM2 {1, −1.721} × {1, 0.1211}	0.0741 −0.0209	0.0950	MM2	7 × 11	BB {1, −1} × {1, −1} MM2 {1, −0.92} × {1, −1}	0.0130 0.0128	0.0002	MM2
3 × 4	MB {1, −0.5} × {1, −1} MM2 {1, −10} × {1, −10}	0.2222 0.0260	0.1962	MM2	7 × 13	BM {1, −1} × {1, −1.2679} MM2 {1, −1 } × {1, −2}	0.0648 0.0400	0.0248	MM2
3 × 5	BM {1, −1} × {1, −1.5} BM {1, −0.77} × {1, −2.18}	0.1600 0.0903	0.0697	MM2	11 × 2	BB {1, −1} × {1, −1} MM2 {1, −1} × {1, −10}	0.0455 0.0090	0.0365	MM2
3 × 7	BB {1, −1} × {1, −1} MM2 {1, −10} × {1, −1}	0.0476 0.0140	0.0336	MM2	11 × 3	BM {1, −1} × {1, −0.5} MM2 [−1, −10]	0.0202 0.0089	0.0113	MM2
3 × 11	BB {1, −1} × {1, −1} MM2 {1, −10} × {1, −1}	0.0303 0.0089	0.0214	MM2	11 × 4	BB {1, −1} × {1, −1} MM2 {1, −1} × {1, −10}	0.2500 0.0168	0.2332	MM2
3 × 13	BM {1, −1} × {1, −1.2679} MM2 {1, −1} × {1, −2}	0.0648 0.0400	0.0248	MM2	11 × 5	BM {1, −1} × {1, −1.5} MM2 {1, −1} × {1, −10}	0.1600 0.0157	0.1443	MM2
4 × 2	BB {1, −1} × {1, −1} MM2 {1, −10} × {1, 0}	0.2500 0.0097	0.2403	MM2	11 × 7	BB {1, −1} × {1, −1} MM2 {1, −1} × {1, −0.92}	0.0130 0.0128	0.0002	MM2
4 × 3	BM {1, −1} × {1, −0.5} MM2 {1, −10} × {1, 0}	0.2500 0.0097	0.2403	MM2	11 × 11	BB {1, −1} × {1, −1} MM2 {1, −1} × {1, −1}	0.0083 0.0083	0	BB
4 × 4	BB {1, −1} × {1, −1} MM2 {1, −10} × {1, −10}	0.3125 0.0437	0.2688	MM2	11 × 13	BM {1, −1} × {1, −1.2679} MM2 {1, −1} × {1, −2}	0.0648 0.0400	0.0248	MM2
4 × 5	BM {1, −1} × {1, −1.5} MM2 {1, −10} × {1, −10}	0.2500 0.0415	0.2085	MM2	13 × 2	MB {1, −1.2679} × {1, −1} MM2 {1, −2} × {1, −9}	0.0648 0.0400	0.0248	MM2
4 × 7	BM {1, −1} × {1, −0.5858} MM2 {1, −10} × {1, −1}	0.2500 0.0264	0.2236	MM2	13 × 3	MM {1, −1.2679} × {1, −0.5} MM2 {1, −2} × {1, −1}	0.0648 0.0400	0.0248	MM2
4 × 11	BB {1, −1} × {1, −1} MM2 {1, −10} × {1, −1}	0.2500 0.0168	0.2332	MM2	13 × 4	BB {1, −1} × {1, −1} MM2 {1, −10} × {1, −2}	0.2500 0.0400	0.2100	MM2
4 × 13	BM {1, −1} × {1, −1.2679} MM2 {1, −10} × {1, −2}	0.2500 0.0621	0.1879	MM2	13 × 5	BM {1, −1} × {1, −1.5} MM2 {1, −2} × {1, −8}	0.1600 0.0400	0.1200	MM2
5 × 2	MB {1, −1.5} × {1, −1} MM2 {1, −10} × {1, −1}	0.1600 0.0096	0.1504	MM2	13 × 7	MB {1, −1.2679} × {1, −1} MM2 {1, −2} × {1, −1.4}	0.0648 0.0400	0.0248	MM2
5 × 3	MM {1, −1.5} × {1, −0.5} MM2 {1, −10} × {1, 0}	0.1600 0.0096	0.1504	MM2	13 × 11	MB {1, −1.2679} × {1, −1} MM2 {1, −2} × {1, −1}	0.0648 0.0400	0.0248	MM2
5 × 4	BB {1, −1} × {1, −1} MM2 {1, −10} × {1, −10}	0.2500 0.0416	0.2084	MM2	13 × 13	MM {1, −1.2679} × × {1, −1.2679} MM2 {1, −1.6} × {1, −2}	0.0690 0.0527	0.0163	MM2
5 × 5	MM {1, −1.5} × {1, −1.5} MM2 {1, −10} × {1, −10}	0.1856 0.0396	0.1460	MM2					

## Data Availability

Data are contained within the article.

## Abbreviations

SL—side lobe; NACF—normalized autocorrelation function; ACF—autocorrelation function; UWB—ultra-wideband; BB—"Barker-Barker”; BM—"Barker-Modification”; MB—"Modification-Barker”; MM—"Modification-Modification” (nested first type codes); MM2—"Modification-Modification 2” (nested second type codes).
